# Optical Genome Mapping Reveals Disruption of the *RASGRF2* Gene in a Patient with Developmental Delay Carrying a De Novo Balanced Reciprocal Translocation

**DOI:** 10.3390/genes15060809

**Published:** 2024-06-19

**Authors:** Rosa Catalina Lederbogen, Sabine Hoffjan, Charlotte Thiels, Ulrike Angelika Mau-Holzmann, Sylke Singer, Maria Viktorovna Yusenko, Hoa Huu Phuc Nguyen, Wanda Maria Gerding

**Affiliations:** 1 Department of Human Genetics, Ruhr-University Bochum, 44801 Bochum, Germany; rosa.lederbogen@rub.de (R.C.L.); sabine.hoffjan@rub.de (S.H.); maria.yusenko@rub.de (M.V.Y.); huu.nguyen-r7w@rub.de (H.H.P.N.); 2 Department of Neuropediatrics, University Children’s Hospital, Ruhr-University Bochum, 44791 Bochum, Germany; charlotte.thiels@rub.de; 3 Institute of Medical Genetics and Applied Genomics, University Tübingen, 72074 Tübingen, Germany; ulrike.mau@med.uni-tuebingen.de (U.A.M.-H.); sylke.singer@med.uni-tuebingen.de (S.S.)

**Keywords:** *RASGRF2*, developmental delay, optical genome mapping, OGM, translocation, balanced chromosomal aberration

## Abstract

While balanced reciprocal translocations are relatively common, they often remain clinically silent unless they lead to the disruption of functional genes. In this study, we present the case of a boy exhibiting developmental delay and mild intellectual disability. Initial karyotyping revealed a translocation t(5;6)(q13;q23) between chromosomes 5 and 6 with limited resolution. Optical genome mapping (OGM) enabled a more precise depiction of the breakpoint regions involved in the reciprocal translocation. While the breakpoint region on chromosome 6 did not encompass any known gene, OGM revealed the disruption of the *RASGRF2* (Ras protein-specific guanine nucleotide releasing factor 2) gene on chromosome 5, implicating *RASGRF2* as a potential candidate gene contributing to the observed developmental delay in the patient. Variations in *RASGRF2* have so far not been reported in developmental delay, but research on the *RASGRF2* gene underscores its significance in various aspects of neurodevelopment, including synaptic plasticity, signaling pathways, and behavioral responses. This study highlights the utility of OGM in identifying breakpoint regions, providing possible insights into the understanding of neurodevelopmental disorders. It also helps affected individuals in gaining more knowledge about potential causes of their conditions.

## 1. Introduction

Balanced reciprocal translocations, characterized by the exchange of segments between two different chromosomes, are relatively common, with an estimated frequency of 0.25% [1]. Although most of these translocations remain phenotypically silent due to the absence of genetic material gain or loss, disruptions in critical genes or regulatory regions can result in significant phenotypic consequences [2,3]. The examination of balanced translocations has been paramount in the discovery of novel genetic disorders, offering insights into the molecular mechanisms underlying various conditions [4,5,6,7].

A crucial aspect of understanding the effects of balanced translocations is the precise resolution of the breakpoint regions. This is important to assess the impact of these genetic rearrangements on gene function and disease pathology. In recent advancements, optical genome mapping (OGM) has emerged as a powerful tool, providing enhanced resolution and clarity in identifying breakpoint regions of chromosomal translocations at the gene level [8].

In this paper, we discuss a case involving a patient with a **de novo** reciprocal translocation, t(5;6)(q13;q23), where optical genome mapping identified the disruption of the *RASGRF2* (Ras protein-specific guanine nucleotide releasing factor 2, NM_006909) gene on chromosome 5. This gene is investigated as a potential candidate for contributing to the patient’s developmental delay. We aim to explore the role of *RASGRF2* through a literature review and assess its implications in relation to our patient’s condition.

## 2. Materials and Methods

### Optical Genome Mapping

The blood samples were collected in EDTA tubes and promptly stored at −40 °C within one day. The extraction of ultra-high-molecular-weight genomic DNA (UMWH gDNA) from white blood cells was performed using the Bionano Prep SP-G2 Frozen Human Blood DNA Isolation Kit according to Bionano Genomics’ instructions (Bionano Genomics, San Diego, CA, USA). Initial quantification of DNA was performed using HemoCue WBC Counter (Radiometer GmbH, Krefeld, Germany). Subsequently, cells were lysed using Proteinase K, and gDNA was bound to a magnetic disc, washed, and quantified once again using a Qubit device (Invitrogen Qubit Fluorometer 3, Thermo Fisher Scientific, Waltham, MA, USA). Following this, the DNA labeling was carried out using the Bionano DLS-G2 labeling kit. The methyltransferase DLE-1 enzyme was employed for the enzymatic labeling approach, specifically targeting the sequence motif CTTAAG within the DNA. The labeled DNA was loaded into the flowcell of the Saphyr chip (Bionano Genomics, San Diego, CA, USA). There, the DNA was linearized through electrophoresis using low-voltage nanochannel arrays on the Saphyr chip. Molecular quality report values were assessed, ensuring a minimum effective coverage of 80×. The run achieved a throughput of 500 Gb. The run showed an average N50 ≥ 150 kbp of 332, an average label density of 15.83 per 100 kbp, and an average mapping ratio of 94.3%. De novo assembly was executed using Bionano Solve software (Version 3.7.2). Analysis was performed using Bionano Access (Version 1.7.2), comparing the results of the sample with the GRCh38/hg38 reference genome map, using the Bionano de novo pipeline. Structural variants with frequencies of 1% or less in the Bionano control population were thoroughly examined. The Bionano control sample SV database comprises 285 ethnically diverse mapped genomes with no reported diseases. Additionally, a detailed analysis of the known reciprocal translocation was conducted.

## 3. Results

### Case Presentation

We present the case of a 10-year-old boy, the only child of non-consanguineous parents, where pregnancy, birth, and early childhood development were unremarkable. However, a significant delay in language development and impairment of fine and gross motor functions became evident during the child’s development, accompanied by borderline intellectual functioning (IQ 80). Clinical findings also included bilateral valgus deformity of the knees, pes planovalgus, and toe walking, as well as, currently, tall stature (>97th percentile) and obesity (BMI 28.5). A psychiatric diagnosis of attention deficit disorder was made. Recently, however, his development has been clearly positive, resulting in a transfer from a special school to a regular school.

Karyotyping revealed a reciprocal translocation in the patient (46,XY,t(5;6)(q13;q23)), which was confirmed by Fluorescence In Situ Hybridization (FISH) and was not detectable in the blood cells of either parent. FISH was performed using the two whole-chromosome painting probes WCP5 (Spectrum Orange) by Vysis and WCP6 (Texas Red) by Cytocell (see Supplementary File S1, for FISH images). Array analysis showed no abnormalities, indicative of a balanced translocation without loss or gain of genetic material. The examination for fragile X syndrome and the panel analysis for syndromic diseases (2021) followed by re-evaluation of exome data (2024) revealed unremarkable findings, apart from variants of uncertain significance in the *SOX5* (rs780885506) and *CIC* (NM_001304815.2, Exon 2: c.2509G>A, p.(Gly837Arg)) genes, which were each carried by an unaffected parent and therefore unlikely to contribute to the patient’s phenotype. Imaging studies, including MRI and EEG, showed no pathological findings. The family history revealed that both the patient’s maternal uncle and the son of the mother’s maternal uncle exhibited signs of intellectual disability in the pedigree analysis; however, no clear diagnosis was made, and these relatives were not available for clinical evaluation or genetic testing.

**Figure 1:**
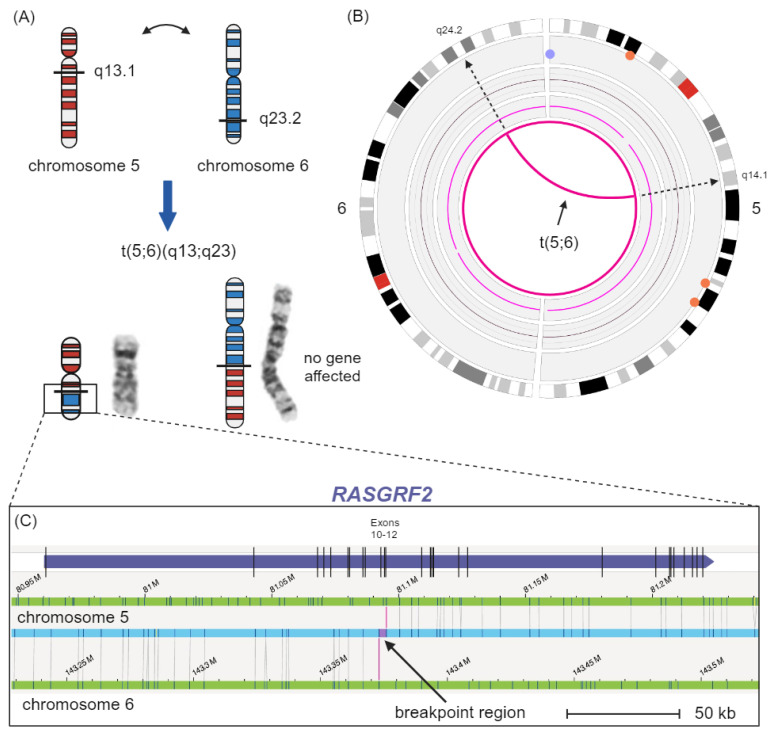
(**A**) Reciprocal translocation t(5;6) results in two new derivative chromosomes in karyotyping, also depicted as ideograms. (**B**) Circos plot results for chromosomes 5 and 6 showing translocation t(5;6) in Bionanogenomics Access software 1.7.2; information from outer to inner rings: cytobands, SV (structural variant) track, CNV (copy number variant) track, VAF (variant allele frequency) segments, and translocations. Orange and purple dots represent additional SVs found with 1% filter (for additional information, see File S2). Dashed arrows show the cytoband in which the breakpoint region is located. Dashed arrows show the cytoband in which the breakpoint region is located. (**C**) Detailed visualization of the structural variant in the *RASGRF2* (Ras protein-specific guanine nucleotide releasing factor 2) gene as observed in detail view.

OGM analysis confirmed the translocation between chromosomes 5 and 6, redefining the breakpoint regions more precisely—from 46,XY,t(5;6)(q13;q23), as identified in karyotyping, to 46,XY,t(5;6)(q14;q24). While the breakpoint region identified on chromosome 6 did not encompass any known gene, this chromosomal rearrangement led to the disruption of the *RASGRF2* gene located on chromosome 5, as shown in Figure 1. 

The exons 10–12 of this gene are located in the breakpoint region. The DLE1 sites surrounding the translocation breakpoint region are located at positions 83,368,100 to 83,371,000. These findings were consistent with preliminary results from conventional chromosome analysis and FISH techniques. Additional structural variants identified by Bionano Access analysis were not associated with our patient’s phenotype according to the current state of knowledge and thus were not considered for further analysis. The identified structural variants can be found in the Supplementary File S2.

## 4. Discussion

The standard diagnostic method for large structural genetic aberrations has primarily been karyotyping, which provides a resolution between 5 and 10 Mb [9]. OGM, as a genome-wide technology, is capable to detect structural aberrations with a significantly higher resolution of 50 kb [10]. In our case, OGM facilitated the resolution of the translocation breakpoint region to the gene level, leading to the identification of our gene of interest, *RASGRF2*. It also led to a more precise redefinition of the breakpoint regions, transitioning from the originally described bands q13;q23 based on classical karyotyping to q14;q24 after OGM analysis. OGM has shown high concordance with standard diagnostic methods [11,12]. For instance, a recent study demonstrated that 9 out of 10 chromosomal translocations previously identified through karyotyping, FISH, or CNV analysis were detected through OGM [8]. The only translocation that was not detected was associated with repetitive sequences on the Y chromosome, underscoring a known limitation of OGM. Additionally, this technology encounters difficulties in analyzing telomeric and centromeric regions [12]. For an even more precise resolution, sequencing technologies like long-read genome sequencing can be utilized, which allow an in-depth analysis down to the base pair level [2]. Nevertheless, in our case, OGM successfully identified the disrupted gene, and additional sequencing would not have offered any extra clinical relevance for our patient.

**Figure 2:**
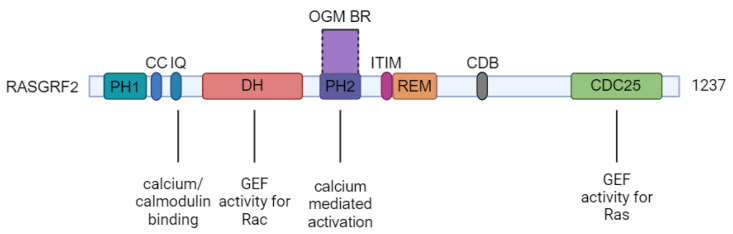
This figure shows the domain structure of the RASGRF2 protein (modified from Nair and Saha et al., 2023 [17]). The domains from left to right are Pleckstrin Homology domain 1 (PH1), Coiled Coil domain (CC), ilimaquinone domain (IQ), Dbl Homology domain (DH), Pleckstrin Homology domain 2 (PH2), Immunoreceptor Tyrosine-based Inhibition Motif (ITIM), Ras Exchanger Motif (REM), Cyclin Destruction Box (CDB), and CDC25 Homology domain (CDC25). The OGM breakpoint region (OGM BR, exons 10–12) is located within the PH2 domain.

The *RASGRF2* gene, disrupted in our patient’s chromosomal translocation t (5;6), has not yet been directly associated with developmental delay in the existing literature. Nevertheless, it is recognized for its vital contribution to neuronal signaling and neurodevelopment. This gene encodes a calcium-regulated guanine nucleotide exchange factor (GEF) that activates GTPases such as Ras and Rac1. These GTPases are integral for coordinating MAP kinase (MAPK) pathways, which are essential for converting external signals into cellular responses, thereby regulating key cellular activities, including proliferation, differentiation, and apoptosis [13,14,15]. Predominantly expressed in neuronal tissues, *RASGRF2* extends over 27 exons and produces an 8100 bp transcript that translates into a 1237-amino-acid protein, characterized by several significant domains, as shown in Figure 2 [16]. 

Among these, the CDC25 domain is instrumental in providing GEF activity for Ras, whereas the DH domain promotes the exchange of GDP for GTP on Rac, indicating a central role in cellular signaling pathways [13,16]. RASGRF2 also has two PH domains (PH1 and PH2). PH1 interacts with membrane lipids in response to receptor stimulation, while PH2 is necessary for calcium-mediated activation. Exons 10–12, which are located in the breakpoint region, are situated within the PH2 domain [17]. The protein’s capacity to bind calcium and interact with calmodulin underscores its crucial function in mediating cellular responses to calcium fluctuations. Besides *RASGRF2*, there is also *RASGRF1*; while highly homologous, they exhibit distinct target specificities and functional roles in signaling pathways related to cell growth, differentiation, and neuronal functions. Notably, *RASGRF1* is implicated in controlling postnatal growth through mechanisms in the hypothalamus, highlighting divergent roles from *RASGRF2* [16].

Analysis within the Decipher database revealed a link between genetic variations in the *RASGRF2* gene and specific phenotypic outcomes. One patient (ID:290543) presented with a heterozygous partial duplication of the *RASGRF2* gene, considered likely pathogenic, extending from the neighboring *MSH3* gene to a region between exon 1 and 2 of *RASGRF2*. This individual showed signs of moderate intellectual disability and obesity. However, there is currently no evidence linking *MSH3* with developmental delay/neurological issues but rather with cancer predisposition [18].

Similarly, another patient (ID: 269277), with a duplication affecting the *RASGRF2* gene, displayed overgrowth and mild global developmental delay, though the pathogenicity of this duplication remains uncertain. According to the Decipher database (available at https://www.deciphergenomics.org/browser, (accessed on 10 June 2024)), there is no information about other genetic variants in these patients. These cases suggest a potential phenotypic spectrum associated with *RASGRF2* gene disruptions, aligning with the clinical presentation observed in our patient. Additional cases (IDs: 326833, 271463, 281046) with uncertain or unspecified pathogenic classifications further reveal associations with phenotypes such as atypical behavior, autism, and overgrowth. 

Functionally, *RASGRF2* is essential in adult neurogenesis, influencing neuron survival and hippocampal functionality, as demonstrated by its crucial involvement in processes such as pattern separation and synaptic plasticity. These functions are integral to the gene’s significant contributions to cognitive mechanisms, highlighting its role in learning and memory [19,20]. Specifically, *RAFGRF2* is essential for initiating long-term potentiation and the enlargement of dendritic spines, key processes in synaptic signaling and neural connectivity [21,22,23]. Additionally, in adult mice, *RASGRF2* is crucial during the initial stages of neuronal differentiation within the dentate gyrus (DG), underlining its critical function in the maturation and integration of new neurons into neural networks [24]. Collectively, these findings underscore the indispensable role of *RASGRF2* in neurodevelopment and synaptic processes, suggesting its significant impact on cognitive functions and potential implications in neurodevelopmental disorders.

Moreover, *RASGRF2* is implicated in various behavioral responses, including drug-related aversive and rewarding behaviors and contextual fear learning [25,26,27]. Certain haplotypes of *RASGRF2* are linked to increased reward sensitivity and higher risk for alcohol use disorders [28]. 

A recent study has identified that specific mutations in the Cavβ2a subunit of voltage-gated calcium channels (VGCCs) are associated with autism spectrum disorder (ASD). The mutations led to aberrant activation of the Ras/ERK/CREB signaling pathway. This pathway, usually regulated by neuronal activity, becomes continuously active with these mutations, altering gene expression and potentially disrupting neuronal development and functionality. *RASGRF2*’s role as a mediator, linking VGCC mutations to abnormal signaling, suggests that disruptions in *RASGRF2* function may contribute to neurodevelopmental disorders [29]. As this example shows, RASGRF2 is an important part of the MAPK pathway. Disruptions not only in VGCCS but also in other components have been shown to lead to neurodevelopmental disorders. ERKs, which are crucial members of the MAPK family, play a role in different forms of learning and memory. Activation of ERK leads to the activation of transcription factors like CREB. Altered CREB signaling has been associated with syndromes which involve intellectual disabilities and developmental delays [30]. Furthermore, *RASGRF2* is hypomethylated among ASD patients, as compared to typically developed individuals, which reinforces its association with neurodevelopmental alterations [31]. Additionally, links between *RASGRF2* and conditions such as schizophrenia and alcohol consumption patterns underscore the gene’s impact on broader neuropsychiatric conditions [32,33]. Earlier research suggested RASGRF2 may not be critical for fundamental neurodevelopment. However, studies have shown that other members of the signaling pathway, such as Rac1, are involved in neuronal migration [34]. Similarly, ERK1/2-deficient mice exhibit impaired neurogenesis with altered cell structures [35]. Additionally, *RASGRF2* directly plays a role in regulating the nuclear migration process during the postnatal development of retinal cone photoreceptors. Mice deficient in *RASGRF2* exhibit ectopic nuclei in the retina, indicating disrupted nuclear positioning [36]. 

In summary, our research emphasizes the crucial role of the *RASGRF2* gene in neurodevelopment, as illustrated by its disruption in our patient. Supported by similar phenotype records from the Decipher database, our findings suggest that *RASGRF2* abnormalities could impact essential neuronal signaling pathways, potentially leading to neurodevelopmental disorders. However, while our data align with existing research, definitive links between our patient’s developmental delay and the specific chromosomal translocation affecting *RASGRF2* require further functional studies. Future research should focus on uncovering the precise molecular mechanisms by which *RASGRF2* disruptions contribute to neurodevelopmental anomalies.

## Data Availability

The data presented in this study are available upon request from the corresponding author due to privacy.

## Supplementary Materials

The following supporting information can be downloaded at: https://www.mdpi.com/article/10.3390/genes15060809/s1. File S1: FISH images of whole-chromosome painting for chromosomes 5 and 6 were obtained using the probes WCP5 (Spectrum Orange) by Vysis and WCP6 (Texas Red) by Cytocell. File S2: Supplementary file listing SVs with 1% filter after original output from bionanogenomics Access software in SMAP format.
