# Supplementary figures and images for: Optical Genome Mapping Reveals Disruption of the RASGRF2 Gene in a Patient with Developmental Delay Carrying a De Novo Balanced Reciprocal Translocation
Source: Genes (Basel). 2024 Jun 19;15(6):809. doi: 10.3390/genes15060809 (PMC11203114; doi:10.3390/genes15060809)

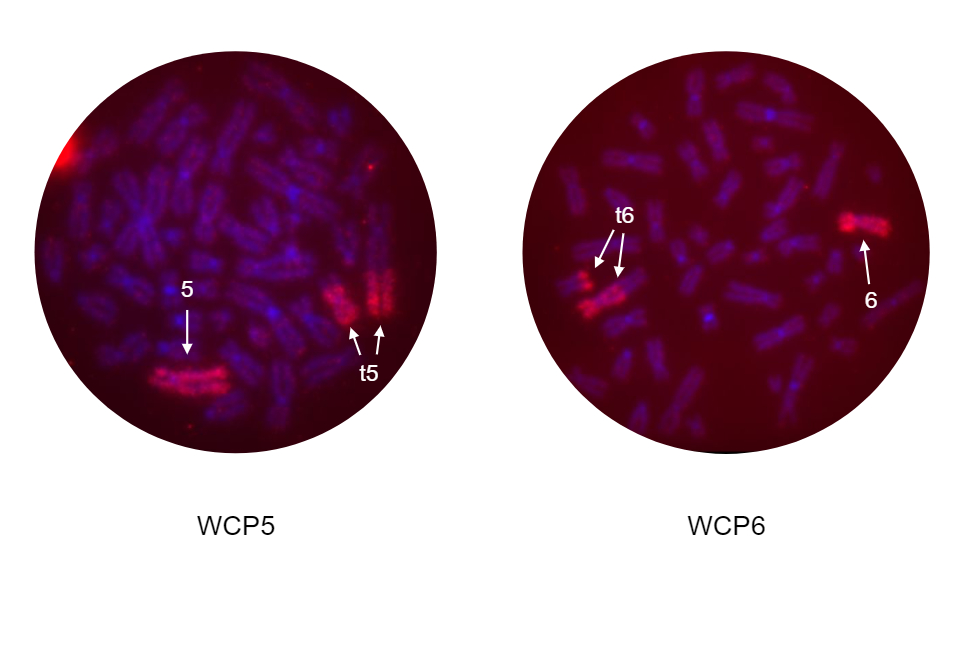

Supplement: Supplementary file 1 [file genes-15-00809-s001.zip › Supplementary File S1.jpeg]
